# Improvement of the Accuracy of InSAR Image Co-Registration Based On Tie Points – A Review

**DOI:** 10.3390/s90201259

**Published:** 2009-02-24

**Authors:** Weibao Zou, Yan Li, Zhilin Li, Xiaoli Ding

**Affiliations:** 1 Shenzhen Institute of Advanced Technology, P.R. China; 2 The University of Southern Queensland, Australia; E-Mails: liyan@usq.edu.au; 3 The Hong Kong Polytechnic University, Hong Kong; E-mails: lszlli@polyu.edu.hk; lsxlding@polyu.edu.hk

**Keywords:** Co-registration, InSAR, interferogram, window size, interval of tie points

## Abstract

Interferometric Synthetic Aperture Radar (InSAR) is a new measurement technology, making use of the phase information contained in the Synthetic Aperture Radar (SAR) images. InSAR has been recognized as a potential tool for the generation of digital elevation models (DEMs) and the measurement of ground surface deformations. However, many critical factors affect the quality of InSAR data and limit its applications. One of the factors is InSAR data processing, which consists of image co-registration, interferogram generation, phase unwrapping and geocoding. The co-registration of InSAR images is the first step and dramatically influences the accuracy of InSAR products. In this paper, the principle and processing procedures of InSAR techniques are reviewed. One of important factors, tie points, to be considered in the improvement of the accuracy of InSAR image co-registration are emphatically reviewed, such as interval of tie points, extraction of feature points, window size for tie point matching and the measurement for the quality of an interferogram.

## 1. Introduction

Interferometric Synthetic Aperture Radar (InSAR) has undergone rapid development since its first proposal by Graham in 1974. Within more than three decades, InSAR has gained increasing attention from researchers in a variety of areas due to its advantages of all weather condition, rapid and accurate topographic data collection [1]. As a result, it has matured to be a widely applied technique in many fields [2], such as surface deformation monitoring [3-5]; forest management and classification [6-9]; ocean current and glacier movement monitoring [10-12]; hydrologic studies [13] and polar research [14,15].

InSAR is a potential technique for generating digital elevation models (DEM) by using the phase component of the complex radar signal [16]. A DEM is one of the most demanded products in the remote sensing community [16]. It is widely used within the geoscientific community, e.g. for mapping purposes, geomorphologic studies based on slope and aspect maps, and as a layer in geographical information systems (GIS) for combining relief data with thematic information. A properly equipped spaceborne InSAR system can be used to produce a highly accurate global DEM with its advantages in significantly less time and at significantly lower cost than other systems [17]. This greatly attracts researchers' attention in military field. In the navigation field, a highly accurate DEM is one of the important information for improving the accuracy of the navigational system [17-21].

InSAR system is used on the Shuttle Radar Topography Mission (SRTM) in February 2000 which is sponsored by the NASA and National Geospatial-Intelligence Agency (NGA) to acquire spatially-continuous elevation information over 80% of the Earth's land mass in a single 10-day Space Shuttle flight [22-26]. The produced DTMs are with 30 m (1 arc-second, SRTM1) and 90 m (3 arc-seconds, SRTM3) grid size and its vertical accuracy ranges from a few meters to decameters [27-28]. It is the first spaceborne single-pass interferometric SAR and will produce the first near-global, high-resolution, digital elevation map. Such a global map will be constructed significantly sooner than with other systems by taking advantage of the unique opportunity offered through augmentation of the previously flown NASA SIR-C and the X-SAR [22].

Since then, a variety of research has being carried out on SRTM data. One kind of research is on the assessment of the accuracy of SRTM DEM. The vertical accuracy of the SRTM Digital Terrain Elevation Data (DTED) level 1 in Southern Greece was assessed by a reference DEM produced from contour lines digitization [23]. Elevations generated by NASA's Laser Vegetation Imaging Sensor (LVIS) were used to assess the validation of SRTM elevations at five study sites of variable relief and land-cover [24]. The results show that there is a linear relationship between the SRTM-LVIS elevation differences and canopy vertical extent [24]. The research carried out by Rodriguez *et al*. documents the results of the SRTM validation effort using global data set generated by SRTM [29]. An accuracy assessment of the C-band SRTM DEM was performed through comparisons with independent elevation data obtained from ICESat and terrestrial height information. It is found that the comparisons can be greatly influenced by dynamic changes of the Earth's surface or instrument-based specifications [30]. Walker *et al*. presented a comprehensive application-specific assessment of the quality of SRTM C-and X-band DEMs in terms of vertical accuracy. The testing results indicated that the quality of SRTM data may be higher than previously thought [25]. It was implemented to evaluate and compare the utility of SRTM and Advanced Spaceborne Thermal Emission and Reflection Radiometer DEMs with baseline DTED-1 topography for mapping lahar flooding hazards from the volcano of Citlaltepetl, Mexico [31]. A comparison of water stages derived from LiDAR, topographic contours and SRTM was implemented. A surprisingly good performance by the SRTM suggests that this is a potentially valuable source for initial flood information extraction in large, topographically homogeneous floodplains [32].

Another is on the comparison between SRTM DEM and other resource derived DEMs. The elevation differences between SRTM C-band 1 and 3 arcsecond resolution DEMs and ICESat 1,064 nm altimeter channel elevation data generated in areas of variable topography and vegetable cover were studied [33]. Geomorphometric comparison between SRTM and the National Elevation Dataset (NED) was implemented to demonstrate how the two sources represent the same surface morphology [34]. The image differences between DEMs are sensitive to even small amounts of misregistration between the two DEMs [35]. The various levels of misregiatration have different impacts on image differences between SRTM and DEMs [35].

Moreover, some research has focused on the applications of SRTM DEMs in many fields. The SRTM C- and X-band DEM data for Ohio and the Amazon were used to analyze the capability of measuring water elevations. The results suggest a great potential for space-based, laterally-spatial measurements of water surface elevations [36]. The accuracy of the Consultative Group for International Agriculture Research (CGIAR) DEM was assessed by comparing elevation values from processed Consultative Group for International Agriculture Research Consortium for Spatial Information (CGIAR-CSI) SRTM data [37]. The SRTM DEM was also used as a reference to provide an adjustment of other elevation data with a precision of five meters [38]. The SRTM data was used to validate the quality of CORONA DEMs [39]. The evaluation of SRTM data was implemented for lahar modeling [40]. An archaeological survey based on the SRTM terrain model was conducted to detect the ancient settlement mounds in the Near East [41]. For many data voids contained in SRTM DEM, a method to fill these voids, based on Triangular Irregular Networks, using a fill surface derived from Russian military maps is presented in [42]. Based on experiments with SRTM data and a few of other data sources, Schetselaar *et al.* presented a data processing and integration methodology of geological field mapping of the LOT 2/LOT 3 project areas and their extensions [43]. A new quantitative topographic algorithm, called HAND (Height Above the Nearest Drainage), based on SRTM DEM was developed. The HAND terrain descriptor produces a normalized digital elevation model that can be applied to classify terrain in a manner that is related to local soil water conditions. This increases usability of the SRTM DEM and provides a new quantitative view on the steady state landscape, one that was missing in the repertoire of terrain descriptors [26].

In short, InSAR is enjoying widespread applications in many fields [3-21]. It is a promising technique to solve some difficult problems in a study area accurately, economically, efficiently and rapidly. There are, however, numerous critical factors affecting the quality of InSAR data. One such factor is the SAR image co-registration that determines both the robustness and the accuracy of InSAR technology and limits its applications and developments. There is no doubt that improving the accuracy of image co-registration in InSAR would greatly benefit the development of InSAR technology.

After this section, the principle of SAR interferommetric processing is introduced. Then, the techniques for improving the accuracy of InSAR image co-registration are described in Section 3, which includes the basic schedule of InSAR image co-registration, determination of tie point interval, extraction of feature points, strategy of tie point matching, determination of window size, selection of transformation model and measurement of the interferogram quality. Finally, a summary is drawn.

## 2. Synthetic Aperture Radar Interferometric Processing

### 2.1. Acquisition of SAR Images

Normally, there are three ways to acquire Synthetic Aperture Radar (SAR) images, according to the number of antenna mounted on the platform and the orientation of the formed baseline [3, 16]: repeat-pass, across-track and along-track. There is no difference on the procedure of InSAR image co-registration regardless of the interferometric way the images are acquired.

**Figure 1.**
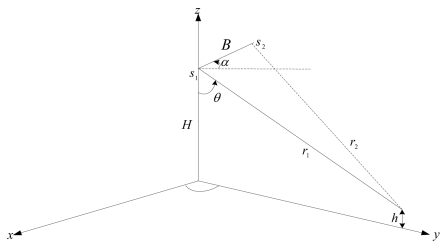
Imaging geometry of SAR interferometry.

In order to understand the principle of SAR image acquisition, the general InSAR imaging geometry is illustrated in Figure 1. Two antennas *s*_1_ and *s*_2_ on ideally parallel flight paths are separated by a baseline *B.* The slant range from antenna *s*_1_ to an illuminated point on the ground is *r*_1_; *r*_2_ is the slant range from antenna *s*_2_ to the same point. With the looking angle *θ*, the angle of baseline with respect to horizontal line α and the flying height *H*, the imaging geometry of InSAR is fixed.

The height can be calculated by the following equations:

(1) $$\sin(\theta -\alpha )=\frac{r_{1}^{2}-r_{2}^{2}+B^{2}}{2r_{1}B}\approx \frac{-\lambda \varphi_{\text{unwrapped}}}{2\pi B}$$

(2) $$\theta =\alpha -\arcsin\left[\frac{\lambda \varphi_{\text{unwrapped}}}{4\pi B}\right]$$

(3) $$h=H-r_{1}\cos\theta$$

where *H* is the antenna *s*_1_ is the height above the reference plane and *h* is the target height. *r*_1_ and *r*_2_ are the slant ranges from antennae to the point on the ground, respectively. *θ* is the looking angle. α is the angle of baseline with respect to horizontal line. *φ_unwrapped_* is the unwrapped interferometric phase. *B* is the baseline. λ is the wavelength of radar signal. Therefore, the DEM data can be derived from radar interferogram.

#### 2.1.1. Acquiring SAR Images by Repeat-pass

**Figure 2.**
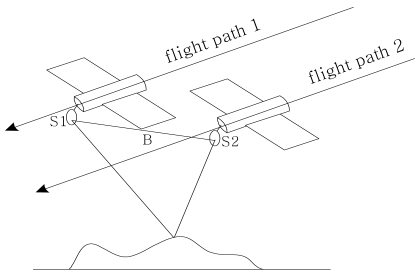
Imaging geometry of repeat-pass SAR interferometry.

The term “repeat-pass” in this paper means to pass the same area repeatedly. In this approach, only one antenna is used and the SAR system acquires SAR data sets by passing the same area twice, covering it with a slightly different viewing geometry. The imaging geometry of repeat-pass SAR interferometry is illustrated in Figure 2. From the figure, it is noticed that, on the first pass, the radar wave is transmitted from antenna *s*_1_, and after interaction with the terrain, the backscattered return is also recorded by antenna *s*_1_. The signal is then processed to a complex SAR image, so does *s*_2_ on the second pass. The signal is processed to another complex SAR image. These SAR images are called the master image and the slave image, respectively, as shown later.

This approach is most suited to spaceborne systems. This is because the precise location of the flight path is required, and satellites typically have much more precise and stable orbital paths in the absence of the atmosphere than aircrafts. Normally, the repeat-pass approach is adopted to obtain SAR images to generate a DEM [44-47]. It is also used to do surface deformation measurements [3-5].

#### 2.1.2. Acquiring SAR Images by Across-track

**Figure 3.**
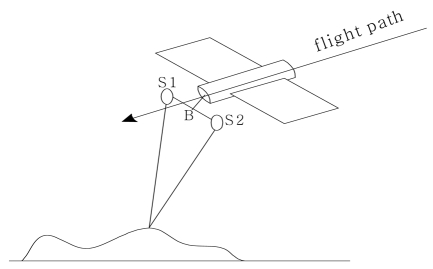
Imaging geometry of across-track SAR interferometry.

Another approach to acquire SAR images is by across-track. Its imaging geometry is illustrated in Figure 3. In this approach, two SAR antennae, *S*_1_ and *S*_2_, are mounted on the same platform. The SAR data are acquired at the same time and the baseline, B, is perpendicular to the flight path. The system, therefore, records the master image and the slave image at the same time but from two slightly different locations.

The main problem with the geometry of the across-track approach in the airborne configuration due to errors caused by the aircraft roll cannot be distinguished from the influence of the terrain slope. This problem is less critical in the spaceborne case, as a satellite track is more stable than an airborne flight path.

The across-track interferometer is also used for DEM generation [48]. In addition, it can also be available for sea surface mapping [49]. A system combining along-track and across track InSAR is carried out for measurement of ocean waves [50].

#### 2.1.3. Acquiring SAR Images by Along-track

**Figure 4.**
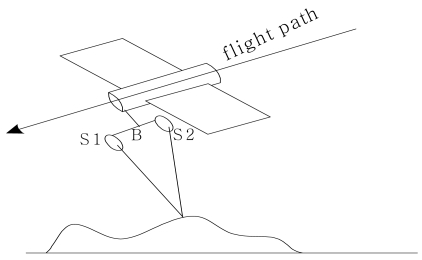
Imaging geometry of along-track SAR interferometry.

The along-track means to acquire SAR images by two SAR antennae, *s*_1_ and *s*_2_. The two antennae follow each other at a short distance on the same orbit and possibly on the same platform for SAR data acquisition. The baseline, *B*, is parallel to the flight direction. Its imaging geometry is illustrated in Figure 4. At present, the along-track approach is only applicable to airborne SAR systems, as it requires two antennae on the same platform. The along-track approach is sensitive to movement of the scatters in the range direction. It can be used to measure Earth-surface velocities. As such, this technique holds promise for the detection of slowly moving ground targets. It was developed in mapping ocean currents. Above sea, it produces a map of the current velocity component parallel to the radar look direction. Now the master image and the slave image are recorded from the same position but at slightly different times. Therefore, the phase differences between the corresponding signals are caused by the movement of the measured object, e.g. water current. The moving surface leads to a Doppler shift according to the phase velocity of the water waves. All stationary targets are not visible whereas the moving ones can be seen in the radar imagery. The along-track mode can be used to measure the velocity of ocean currents and on-ground objects [51,52].

The quality of interferometric measurements depends on the relative distance of the antenna(s), the so-called base line. Across-track and along-track are only possible using airborne systems with two antennae. The yaw and pitch cause baseline components in y- and z-direction produce additional phase differences. The base line can, therefore, be readily found to the required precision. In principle, repeat-pass way is also possible for aircraft, but the flight track and the aircraft attitude should be known with sufficient accuracy.

### 2.2. Interferometric Processing of SAR Images

**Figure 5.**
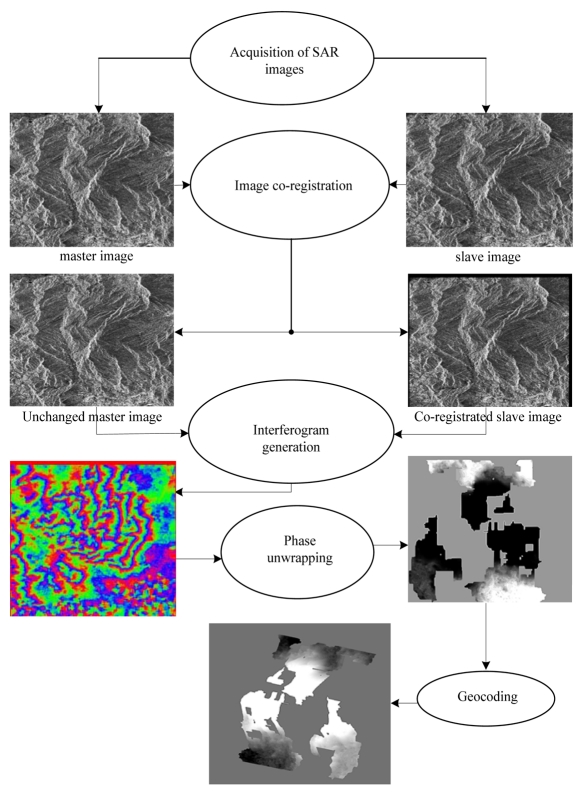
SAR image processing procedures.

There are five steps in the procedures for processing InSAR images. They can be summarized as shown in Figure 5 as follows:

1. Acquisition of SAR images;
2. Co-registration of two SAR images;
3. Generation of interferogram;
4. Phase unwrapping of the wrapped interferometric phase;
5. Geocoding of DEM.
6. DEM is the final product of InSAR image processing.

## 3. Procedures of InSAR Image Co-registration

### 3.1. Co-registration of InSAR Image

A lot of research has been carried out to reduce the errors that are caused by interferometric processing. The InSAR processing consists of co-registration, interferogram generation, phase unwrapping and geocoding. Research has been carried out on co-registration [44,45,53-56], generation of interferograms [44], phase unwrapping [58-61], and geocoding [62].

The co-registration of SAR complex images is the first step in interferometric processing and it is one of the most important processing procedures involved in InSAR. For the quality of the final products, accurate co-registration of the two input images is a prerequisite. Based on a good image registration, ideally up to 1/10 of a pixel, interferometric products with reliable quality could be achieved.

**Figure 6.**
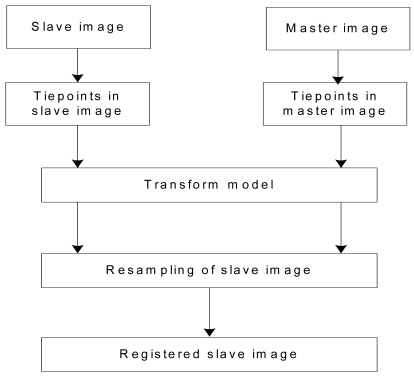
Procedures of InSAR image co-registration.

**Figure 7.**
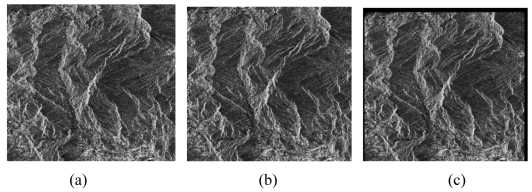
A pair of SAR images (a) and (b) and its co-registered slave image. (a) a master image; (b) a slave image; (c) a co-registered slave image.

The SAR image co-registration chain consists of two steps: i.e., coarse co-registration and fine co-registration. The fine co-registration is an important step, which includes selection of tiepoints, tiepoint matching, transformation models and resampling method shown in Figure 6. A pair of SAR images and its co-registered slave image are shown in Figure 7, respectively. The distribution and the window size of tie points for image co-registration will greatly affect the final generation of SAR image co-registration. Some researches have been implemented on these aspects [44,45,53-56].

An automatic approach with multi-step image matching algorithm was proposed by Liao [63]. In the first step, some interesting tie points have been selected by an operator in a grid form in the master image. The grid size is 9 × 9. The tie point matching was carried out based on a normalized correction coefficient. A set of window sizes during tie point matching is adopted to do experiments. Good results of InSAR image processing are obtained based on the window size, 63 × 63. Therefore, it was selected as the optimum window size for tie point matching. The next step based on relaxation technique is used to ensure the reliability. At last, least-squares adjustment is carried out to improve the accuracy of co-registration. The multi-step strategy is recommended as the first selection for image co-registration.

Another method for image co-registration in the Fourier domain, using Fast Fourier Transform (FFT), is performed [64]. Indeed, this method works very fast. However, it is just suitable for the stationary regions. For non-stationary regions, the result is unreliable.

There are three kinds of issues in image co-registration. The first is the selection of tie point that includes two factors: the distribution of tie points; and the number of tie point to be selected. For the distribution of tie points, theoretically, more tie points will result in a more reliable registration. It has been stated in many literatures that tie points with features should be selected and evenly distributed over the whole image. However, too many tie points would result in a dramatic increase in calculation but it does not necessarily result in a more reliable co-registration. The second is the tie point matching that includes two factors: the window size, and the method for matching. The third is the mathematical models for transformations. They will be described in the following sub-sections, respectively.

### 3.2. Determination of Interval of Tie Points for Co-registration

Conventionally, tie points are usually selected in a grid form, i.e. at grid nodes. Tie points grid sizes of 2×10, 9×9, 6×6 and 8×8 have been adopted by Zebker *et al*. [1], Wan [65], Yang and Wang [66] and Liu *et al*. [67], respectively. At present, the selection of tie points is determined mainly by experience.

The interval of tie points greatly influences the quality of image co-registration and the accuracy of final product, DEM. The optimum interval of tie points for image co-registration is systematically conducted [44, 68]. In the study, only the interval of tie points is considered while other factors are kept unchanged. Four pairs of SAR images of 1,760×400 pixel size of the Hong Kong area were used for testing. The effects of interval of tie points are assessed by a relative measure for the quality of the resultant interferogram and an absolute measure for the accuracy of the resultant DEM. Results show that the effect of tie point interval on the accuracy of the final DEM is not linear. When the tie point interval is smaller than 273×44 pixels (273 pixels in row and 44 pixels in column), the variation in the resultant DEM accuracy is not significant. It is also noticeable that an interval of 205×34 pixels always results in the best or very good results. An interval of around 200×30 pixels (200 pixels in row and 30 pixels in column) is an appropriate choice for the selection of tie points for image co-registration in hilly areas.

### 3.3. Extraction of Feature Points for Tie Point Matching

**Figure 8.**
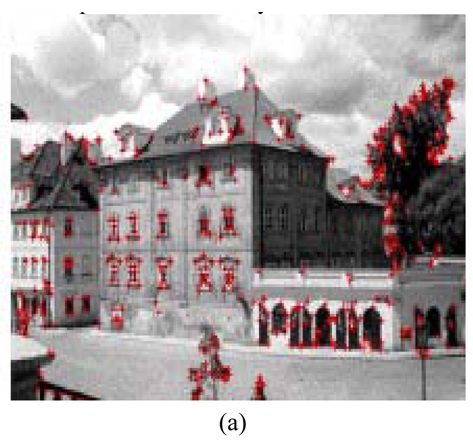

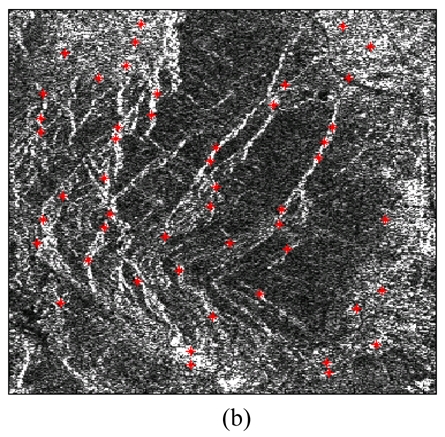
Feature points extracted by different ways. (a) feature points extracted by Interest Operators; (b) feature points extracted by wavelet transform.

Generally, the points with special features, such as intersection and airports, are possibly selected as tie points and if feature points evenly distributes in the image will improve the reliability of co-registration. However, such feature points can not always be found in an image. Therefore, the connotation of feature points should be enlarged. Some methods have been used to extract feature points from images. One of the methods is Interest Operators [69], which is based on the maximum gradient to extract feature points as shown in Figure 8(a). However, each pixel gradient is calculated based on the original whole image. Therefore, too much calculation is needed.

Wavelets can also be used to extract feature points from images [70]. Therefore, an enlarged connotation of feature points in wavelet domain for InSAR image co-registration is developed by Zou [71]. A method to extract feature points with wavelet transform to serve as tie points for co-registration is implemented shown in Figure 8(b). The extraction of feature points is based on the wavelet gradient modulus maximum. Compared with Interest Operators, with wavelets the wavelet gradient is implemented not in the original image but at its highest level so that it reduces the complexity of computation and makes the processing fast. In order to make the co-registration more reliable, the feature points extracted by wavelet and the points selected in grid form are combined together used as tie points [71]. Good experimental results have been achieved. Therefore, this can be considered as a recommended operation for feature extraction.

### 3.4. Strategy for Tie Point Matching

The second issues in image co-registration should be considered is the tie point matching that includes two factors: the window size, and the strategy for matching. Generally, there are two kinds of methods for tie point matching: one is based on spectral maximization; another is the image cross-correlation maximization [72].

For the first method, a small window centered around the tie point in the master image is selected, and it is moved on the slave image. When the peak of the Fourier Transform of the product of the master image multiplied by the complex conjugate of the slave image is maximized, the matching is regarded as the best one. A large amount of calculation is carried out in the method.

About the second method, the cross-correlation coefficient is used as a parameter for evaluating the matching results. The principle is that given a window of pixels on the master image, there will be many possible matching windows of the same size, but the one matching with the highest cross-correlation coefficient is regarded as the best matching. The central pixels of the two windows are then regarded as the corresponding image points found.

Let *z*_1_(*m*, *n*) and *z_2_*(*m*, *n*) represent the two SAR complex images:

(4a) $$z_{1}(m,n)=a_{1}(m,n)e^{j\varphi_{1}(m,n)}$$

(4b) $$z_{2}(m,n)=a_{2}(m,n)e^{j\varphi_{2}(m,n)}$$

where *m* = 0, 1, 2, Λ, *M*-1, *n* = 0, 1, 2, Λ, *N*-1, and the size of the image is *M*×*N*. The cross-correlation coefficient γ is expressed as:

(5) $$\gamma =\frac{{\left|E(z_{1}z_{2}^{*})\right|}^{2}}{\sqrt{E({\left|z_{1}\right|}^{2})E({\left|z_{2}\right|}^{2})}}$$

The expanded form of equation (5) can be written as:

(6) $$\gamma (m,n)=\frac{\left|\sum_{l=-(L-1)/2}^{(L-1)/2}\;\sum_{k=-(K-1)/2}^{(K-1)/2}z_{1}(m+l,n+k)z_{2}^{*}(m+l,n+k)e^{-j\varphi (m+l,n+k)}\right|}{\sqrt{\sum_{l=-(L-1)/2}^{(L-1)/2}\;\sum_{k=-(K-1)/2}^{(K-1)/2}{\left|z_{1}(m+l,n+k)\right|}^{2}\;\sum_{l=-(L-1)/2}^{(L-1)/2}\;\sum_{k=-(K-1)/2}^{(K-1)/2}{\left|z_{2}(m+l,n+k)\right|}^{2}}}$$

where *L*×*K* is the image patch size, *φ*(*m*, *n*) is the phase difference between two images and * denotes the complex conjugate. It can be imagined that the computation will be heavy due to the involvement of φ. In order to reduce the computation, an alternative solution can be carried out, making use of the power correlation coefficient *ρ̂* [56]. The computation of *ρ̂* is as follows:

(7) $$\hat{\rho }\;(m,n)=\frac{\left|\sum_{l=-(L-1)/2}^{(L-1)/2}\;\sum_{k=-(K-1)/2}^{(K-1)/2}{\left|z_{1}(m+l,n+k)\right|}^{2}{\left|z_{2}(m+l,n+k)\right|}^{2}\right|}{\sqrt{\sum_{l=-(L-1)/2}^{(L-1)/2}\;\sum_{k=-(K-1)/2}^{(K-1)/2}{\left|z_{1}(m+l,n+k)\right|}^{4}\;\sum_{l=-(L-1)/2}^{(L-1)/2}\;\sum_{k=-(K-1)/2}^{(K-1)/2}{\left|z_{2}(m+l,n+k)\right|}^{4}}}$$

The relation between *γ̂* and *ρ̂* is as follows:

(8) $$\hat{\gamma }=\left\{ \begin{array}{l} \sqrt{2\hat{\rho }-1}\;\hat{\rho }\;>\;0.5\\ 0\;\hat{\rho }\leq 0.5 \end{array}\right.$$

However, because of the influence of noise, correlation coefficient array maybe has minimax values and maximum correlation coefficient is out of searching window sometimes [73]. So a new correlation coefficient is defined to settle the problem, in which the key point is to form a section of 4×4 pixels. The equation of correlation coefficient is as follows:

(9) $$\gamma {(m,n)}_{\text{new}}=\frac{\sum_{m=1}^{M/4}\;\sum_{n=1}^{N/4}\frac{\left|\sum_{m=1}^{4}\;\sum_{n=1}^{4}Z_{1}(m,n){Z_{2}}^{*}(m,n)\right|}{\sqrt{\sum_{m=1}^{4}\;\sum_{n=1}^{4}{\left|Z_{1}(m,n)\right|}^{2}\;\sum_{m=1}^{4}\;\sum_{n=1}^{4}{\left|Z_{2}(m,n)\right|}^{2}}}}{\frac{M}{4}\;\frac{N}{4}}$$

### 3.5. Determination of Window Size for Tie Point Matching

The window size is another important factor to be considered for tie point matching, which can greatly affect the result of tie point matching. Too large a window size would result in not only a dramatic calculation but also the appearance of pseudo-tie point. However, too small a window size would make the co-registration unreliable. A multi-window sized 21×21 pixels based on maximum cross-correlation is developed by Li and Fan [74]. The window size, 33×33 pixels, 9×9 pixels and 43×43 pixels have been adopted by Zebker *et al*. [55], Weydahl [53] and Liao [63]. However, no theory has been developed to guide the selection of window size.

**Figure 9.**
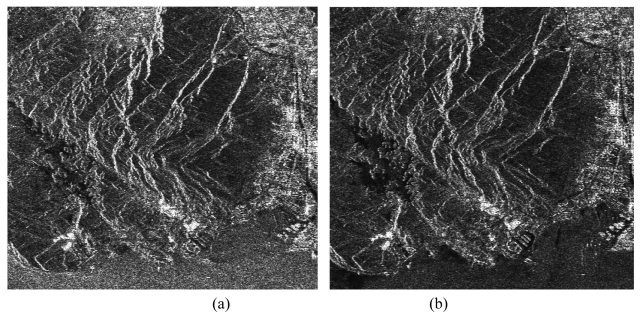
A pair of SAR images (Tai Lam in Hong Kong). (a) the master image; (b) the slave image.

**Figure 10.**
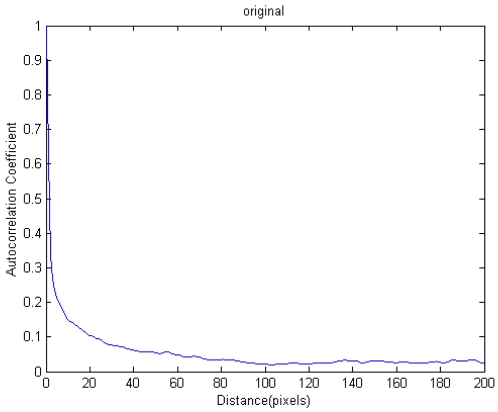
Auto-correlation coefficient of the Tai Lam image.

Recently a method for automated determination of an optimum window size for tie point matching was presented [45], which aims to find a mathematical method to determinate an optimum window size. The method is based on the decomposition of auto-correlation of the SAR image with wavelet transform to select an optimum window size for tie point matching. In this method, the auto-correlation of the SAR images, which reflects the similarity between image pixels, is used as a basis for analysis. To measure such a similarity, auto-correlation coefficient is the most widely used measure. It is defined as:

(10) $$R(d)=\frac{\text{Cov}\;(d)}{V}$$

where *R*(*d*) is the auto-correlation coefficient of all pixels with a distance of *d* part; *Cov*(*d*) is the covariance of all pixels with a distance of *d* part, and *V* is the variance of all pixels. Respectively, they are as follows:

(11) $$V=\frac{\sum_{i=1}^{m}\sum_{j=1}^{n}{\left(\left|Z_{i,j}\right|-K\right)}^{2}}{m\;n-1}$$

(13) $$\text{Cov}(d)=\frac{\sum_{i=1}^{m}\;\sum_{j=1}^{n}\left[\left(\left|Z_{i,j}\right|-K)\;(\left|Q_{i,j+d}\right|-K\right)\right]+\sum_{i=1}^{n}\;\sum_{j=1}^{m}\left[\left(\left|Z_{i,j}\right|-K)\;(\left|Q_{i+d,j}\right|-K\right)\right]}{2\;m\;n-1}$$

and

(14) $$K=\frac{\sum_{i=1}^{M}\;\sum_{j=1}^{N}\left|Z_{i,j}\right|}{M\;N}$$

where *m* = 0, 1, 2, Λ, *M*-1, *n* = 1, 2, Λ, *N*-1, and the size of the image is *M*×*N; Z_i_*_,_*_j_* is one pixel with a coordinate (*i*, *j*) (expressed in pixel) in the image, its grey value is |*Z_i_*_,_*_j_*|; *Q_i_*_,_*_j+d_* is another pixel with a distance *d* apart from the pixel *Z_i_*_,_*_j_* in column direction, |*Q_i_*_,_*_j+d_*| is its gray value; *Q_i+d_*_,_*_j_* is another pixel with a distance *d* apart from the pixel *Z_i_*_,_*_j_* in row direction, |*Q_i+d_*_,_*_j_*| is its gray value; *K* is the average grey value of all pixels. A pair of SAR images and its curve of auto-correlation coefficient are shown in Figures 9 and 10, respectively.

**Figure 11.**
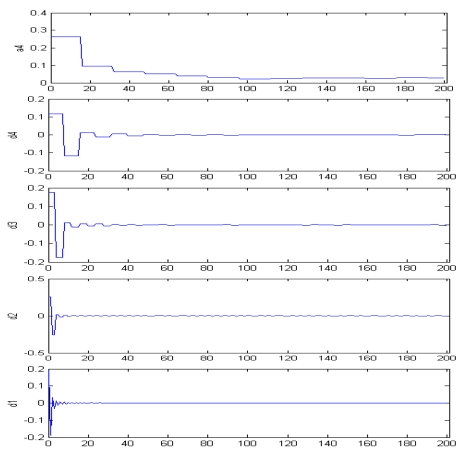
Components of the auto-correlation of the Tai Lam image decomposed by wave db1. *d*_1_ ∼ *d*_4_ are the high-frequency components and *a*_4_ is the lowest-frequency component.

Then, the auto-correlation function is decomposed into waves of various frequencies by wavelet transform as shown in Figure 11. By a combined analysis of the variations of wave amplitudes with frequency, the optimum window size for tie point matching can be determined. When wavelet db1 is used to decompose the auto-correlation coefficient, the jumping point (on the lowest-frequency component), at which the percentage amplitude is smaller than 15% of the amplitude at *d* = 0 and the difference of amplitude in percentage is smaller than 10%, can be selected as the optimum window size. In this way, an optimum window size could be determined for any pair of SAR images.

### 3.6. Selection of Transformation Models

Another issue for image co-registration are the mathematical models used for transformations. The transformation models can also affect the reliability of co-registration. In InSAR, two complex images are used, which cover the same scene but are taken from slightly different positions. Geometrically, they are in different coordinate systems. Their orientations could also be quite different. Therefore, an operation is required to bring them down to an identical coordinate system so as to have an identical orientation. There are two possible solutions: either to bring both down to an absolute ground coordinate or to fit one image into the coordinate system of the other. Normally, co-registration refers to the latter. In order to bring one image to fit into the coordinate system of the other, the relationship between these two images needs to be established. In InSAR practice, some sorts of polynomials are used as approximate models for the transformation between them. The models include geometric transformation models, and radiometric transformation models for re-sampling.

In order to enhance the accuracy of the results, different transformation models could be adopted in different situations. In the case of a photograph, one frame of an image is taken at one time. For each frame, there are six orientation elements (three rotations and three translations). The elements are the same for all image pixels on the same frame. If the image coordinate system is that the origin being the center of the image and the x-axis being along the flight direction, an arbitrary Cartesian coordinate system could possibly be used in the image plane. It is as follows [75]:

(14a) $$x=\frac{A_{1}X+A_{2}Y+A_{3}Z+A_{4}}{A_{9}X+A_{10}Y+A_{11}Z+1}$$

(14b) $$y=\frac{A_{5}X+A_{6}Y+A_{7}Z+A_{8}}{A_{9}X+A_{10}Y+A_{11}Z+1}$$

where, *A*_1_∼*A*_11_ are the coefficients; *x* and *y* are the coordinates of an image point; *X*, *Y* and *Z* are the coordinates of its position on the ground.

In the case of scanner images, image pixels are acquired scan by scan. A frame of an image may consist of thousands of scan lines. In this case, there are six orientation elements and there is a need of at least three control points for the computation of coefficients for each scan line. This is not feasible. The problem could be simplified if the relationship among the scan lines is known. However, this is normally not the situation. In this case, a single polynomial could be used to approximate the geometric model transformation for all scan lines [75]:

(15a) $$x=a_{0}+a_{1}X+a_{2}Y+a_{3}X^{2}+a_{4}Y^{2}+a_{5}XY+\cdots$$

(15b) $$y=b_{0}+b_{1}X+b_{2}Y+b_{3}X^{2}+b_{4}Y^{2}+b_{5}XY+\cdots$$

where, *a*_0_∼*a*_5_ and *b*_0_∼*b*_5_ are the coefficients; *x* and *y* are the coordinates of an image point; *X* and *Y* are the coordinates of its position on the ground. Indeed, in this case, the errors caused by distortions are re-distributed rather than removed by the polynomial function.

The latter case is suitable for InSAR practice. There are several functions used as the polynomial. They are known as nearest-neighbor interpolation, bilinear interpolation, and bicubic interpolation [76]. The bicubic function is adopted as the polynomial for the transformation between the master and slave images [44, 45, 68]. In InSAR image co-registration, (*x*, *y*) is the pixel's coordinate in the slave image while (*X*, *Y*) is the pixel's coordinate in the master image.

Zou *et al*. presented a method for an accurate co-registration of spacebore repeat-pass InSAR image based on Matrix Transformation, which considers the offsets errors generated by sensor trajectory excursion in repeat-pass style [77]. The coordinate transformation between the master image and the slave image is implemented by a matrix in which both shifting and rotating procedures are carried out.

### 3.7. Measurement for Quality of an Interferogram

The quality of an interferogram is an indicator for the reliability of SAR image co-registration. So far, the root mean square error (RMSE) of the residuals at tie points after least-squares adjustment was adopted as a measure for the quality of an interferogram. It implies that the smaller the RMSE, the better the interferogram. However, it is not always the case. In some cases, when the RMS is small, the resultant interferogram is not so good [68].

From the literature, it can be found that visual appearance of the fringes is widely used to indicate the quality of interferograms [68,78], that is, clear fringes imply good quality of an interferogram. In other words, if the interferometric fringes can be observed clearly, the quality of interferogram must be good. On the other hand, the blurry fringes will be found in bad interferogram. Visual inspection is a kind of manual interpretation.

**Figure 12.**
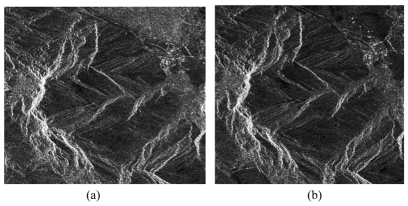
A pair of SAR images. (a) the master image; (b) the slave image.

**Figure 13.**
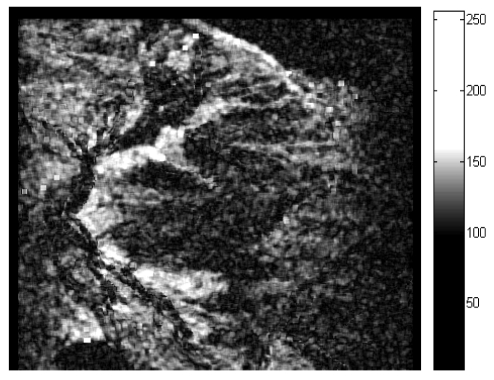
The corresponding coherence image.

The coherence of interferometric SAR is another indicator for the quality of interferogram [16,56]. The coherence between two SAR images can be theoretically defined on a pixel basis as the correlation coefficient and varies in the range of 0 to 1. It significantly influences the accuracy of phase in interferogram. So the degree of coherence can be used as a quality measure for the interferogram. The stronger the coherence, the better the quality. The coherence of an image varies from area to area or even from pixel to pixel. Therefore coherence image is produced to show the quality variation of an interferogram over the entire area. Figure 12 is a pair of SAR image and its corresponding coherence image is shown in Figure 13 [79]. In this image, some areas are brighter while some areas are darker. The brighter areas correspond to good coherence and the dark areas correspond to bad coherence.

To make a coherence measure quantitative, histogram of the coherence image is used. More precisely, the mean of the distribution and the standard deviation (STD) from the mean are the two quantitative parameters for the coherence. However, it is found even though the coherence images are quite different and the distributions of the pixel number in different interval of coherence are quite different, the actual values for the means and STD can be quite similar [79]. Therefore, such statistical parameters are not sufficient to differentiate the quality of interferograms. Based on the above, Li *et al*. [79] has proposed a more reliable quantitative measure for the InSAR interferogram based on the “sum of phase differences” (SPD).

The SPD method is based on the characteristics of the interferogram. Because an interferogram is generated by means of the product of the master image and the complex conjugate of the registered (slave) image, it contains an interferometric phase. Different colour fringes express different phase values in the interferogram. In an ideal case, the phase value changes gradually from 0 to 2π radians. The value of the phase difference between two pixels that are in neighboring fringes should be infinitesimal. If the two pixels are in the same colour fringe, the phase difference between them should be zero. Therefore, the smaller the SPD, the better the quality of the interferogram.

The *SPD_local_* of one pixel is calculated as follows [79]:

(16) $${\text{SPD}}_{\text{local}}(x,y)=\frac{1}{8}\;\sum_{l=-1}^{1}\;\sum_{k=-1}^{1}\left|\varphi_{\text{flattened}}\;(x,y)-\varphi_{\text{flattened}}\;(x+l,y+k)\right|$$

where *x* = 0,1,2, ⋯, *p* − 1; *y* = 0,1,2, ⋯, *q* − 1 and the interferogram image size is *p* × *q*.

The *SPD_whole_* of the whole interferogram is obtained by summarizing the *SPD_local_* of all pixels as follows:

(17) $${\text{SPD}}_{\text{whole}}=\sum_{x=0}^{p-1}\;\sum_{y=0}^{q-1}{\text{SPD}}_{\text{local}}(x,y)$$

The indicator is adopted to measure the quality of the interferogram by Zou [71,80].

## 4. Summary

This paper aims to provide researchers with more information about the achievements in the study of InSAR image co-registration. Co-registration is the first and one of the most important steps in the interferometric InSAR data processing. In this step, the distribution of tie points, the size of window for tie point matching, extraction of feature points and the measurement for the quality of an interferogram can affect the accuracy of the co-registration, which in turn determines the quality of any InSAR products, and thus the quality of the final InSAR product, e.g. DEM. Therefore, the enhanced techniques on these critical factors for improving the accuracy of InSAR image co-registration with tie points are reviewed in this paper. Specially, the most recent achievements on extraction of feature points, effects of interval of tie points, determination of an optimum window size and an indicator of an interferogram quality are emphatically reviewed.
